# Effects of sodium/glucose cotransporter 2 inhibitors on the coagulation profile in patients with coronary-artery disease and type 2 diabetes mellitus: a retrospective cohort study

**DOI:** 10.3389/fcvm.2025.1588797

**Published:** 2025-05-07

**Authors:** XiaoYue Qin, GuoBin Song

**Affiliations:** Department of General Practice, Shijiazhuang People’s Hospital, Shijiazhuang, China

**Keywords:** sodium/glucose cotransporter 2 inhibitors, coronary-artery disease, type 2 diabetes mellitus, coagulation profile, activated partial thromboplastin time

## Abstract

**Aims:**

Patients with coronary-artery disease (CAD) and type 2 diabetes mellitus (T2DM) are often in a hypercoagulable state and have an increased thrombosis risk. We aimed to evaluate the effects of sodium/glucose cotransporter 2 inhibitors (SGLT2is) on coagulation function and explore their potential role in regulating coagulation in these patients.

**Methods:**

We conducted a retrospective cohort study between June 2020 and June 2024 in patients with CAD and T2DM. Eligible patients were assigned to either the SGLT2i or non-SGLT2i group. Clinical information, laboratory tests, and echocardiographic (EKG) examination results were retrieved. We performed inter- and intragroup comparisons of coagulation function measurements before and after treatment, and also conducted regression analysis to assess the impact of treatment on coagulation function.

**Results:**

A total of 121 patients were included, with 49 and 72 in the SGLT2i and non-SGLT2i groups, respectively. After 30 days of treatment, antithrombin III (AT-III) activity increased by 5.39% (*P* = 0.026) in the SGLT2i group, but slightly decreased in the non-SGLT2i group. SGLT2 is also decreased D-dimer levels by 95 mg/L (group *P* = 0.051, group:time *P* = 0.075). Further regression analysis showed a significant interaction between group and time for AT-III and D-dimer (*P* = 0.026 and *P* = 0.039). Additionally, prothrombin time (PT) showed a slight increase after SGLT2i treatment.

**Conclusion:**

SGLT2is could affect coagulation function by prolonging coagulation time and increasing anticoagulatory activity in patients with T2DM and CAD. These drugs could be used to ameliorate hypercoagulable states and reduce thrombosis risk in these patients.

## 1 Introduction

Type 2 diabetes mellitus (T2DM) is a common disease. It is reported that up to 34.8% of T2DM patients have comorbid coronary-artery disease (CAD) (1). The presence of both CAD and T2DM can activate intrinsic- and extrinsic-coagulation pathways, elevate factor VIII (FVIII) levels, shorten activated partial thromboplastin time (APTT), and reduce the activity of anticoagulant proteins [protein C, protein S, and antithrombin (AT)], leading to a sustained hypercoagulable state and a high risk for thrombosis (2–4). Appropriate anticoagulant and antiplatelet treatments are required to effectively prevent thrombotic formation and cardiovascular (CV) complications in these patients with multiple comorbidities.

Sodium/glucose cotransporter 2 inhibitors (SGLT2is) are a class of antihyperglycemic agents. In addition to their glucose-lowering effects, SGLT2is can provide CV protection and reduce the incidence of major adverse cardiovascular events (MACEs) (5–7). These drugs are recommended in patients with T2DM and CAD to mitigate their CV risks (8). Several studies have demonstrated that SGLT2is can increase adenosine monophosphate (AMP)–activated protein kinase activity and enhance phosphorylation of endothelial nitric oxide synthase (eNOS) to directly regulate vascular homeostasis (9–11). In addition, the anti-inflammatory activities of SGLT2is alleviate endothelial inflammation and maintain endothelial integrity (12, 13). A recent study reported that SGLT2is could reduce mortality caused by CV disease (CVD), probably via their thrombosis-preventing effects (14). All of this evidence suggests a potential role for SGLT2is in modulating thrombosis, which might be beneficial to patients in hypercoagulable states. However, clinical research into the anticoagulatory role of SGLT2is in patients in hypercoagulable states, such as those with comorbid CAD and T2DM, is limited.

Therefore, we performed a retrospective study aiming to assess the effect of SGLT2is on coagulation function in patients with CAD and T2DM. Our objective was to provide an evidence-based strategy to optimize anticoagulant therapy, decrease disease complications, and improve quality of life (QoL) in these patients.

## 2 Methods

### 2.1 Study design and participant selection

This was a retrospective cohort study in patients who visited Shijiazhuang People's Hospital (Shijiazhuang, China) between June 2020 and June 2024. The study protocol was approved by the hospital's ethics committee (Approval No. 083), and the study was conducted in compliance with the ethical standards of the Declaration of Helsinki. Informed consent was waived due to the retrospective study design.

Inclusion criteria were patients (1) aged 18–90 years; (2) diagnosed with CAD based on typical angina symptoms, coronary angiography (CAG), and/or echocardiographic (EKG) results; and (3) with a diagnosis of T2DM per World Health Organization (WHO) criteria. Exclusion criteria were patients (1) using SGLT2is for at least 1 month before study initiation; (2) with severe hepatic or renal impairment [defined as alanine aminotransferase (ALT) or aspartate aminotransferase (AST) >3× the upper limit of normal, or an estimated glomerular filtration rate (eGFR) <30 ml/min/1.73 m^2^]; (3) comorbidly using other antidiabetic agents [glucagon-like peptide-1 (GLP-1) receptor agonists] during the study period; (4) who were pregnant or lactating; (5) with malignancies, hematological disorders, or infections; (6) on long-term anticoagulant therapy (warfarin or heparin); (7) with psychiatric disorders, cognitive impairments, or any other conditions compromising their ability to follow the treatment regimen; or (8) who were allergic to SGLT2is and therefore discontinued the treatment. Patients with incomplete or missing data were also excluded.

### 2.2 Data collection

We reviewed patients' medical records and retrieved the following information: age, sex, height, weight, body mass index (BMI), systolic blood pressure (SBP), and diastolic blood pressure (DBP). Medications, including SGLT2is (dapagliflozin or empagliflozin 20 mg 1×/day), aspirin, clopidogrel or ticagrelor, statins or ezetimibe, angiotensin-converting enzyme inhibitors (ACEIs) or angiotensin II receptor blockers (ARBs), angiotensin receptor neprilysin inhibitors (ARNIs), beta blockers, diuretics, metformin, sulfonylureas, and insulin, were recorded.

We documented laboratory test results before and 1 month after SGLT2i treatment. Tests included coagulation markers such as APTT, antithrombin III (AT-III), prothrombin time (PT), prothrombin activity (PTA), international normalized ratio (INR), thrombin time (TT), fibrin degradation products (FDPs), and D-dimer. We also tested for fasting glucose (Glu), glycated hemoglobin (HbA1c), red blood cell count (RBC), hemoglobin (HGB), hematocrit (Hct), mean corpuscular volume (MCV), total platelet count (PLT), plateletcrit (PCT), mean platelet volume (MPV), low-density lipoprotein (LDL), high-density lipoprotein (HDL), apolipoprotein A1 (ApoA1), apolipoprotein B (ApoB), total cholesterol (TC), triglycerides (TG), lipoprotein a [Lp(a)], ALT, AST, cholinesterase (ChE), alkaline phosphatase (AKP), gamma-glutamyl transferase (GGT), urea, uric acid (UA), and eGFR. Cardiac function was evaluated by EKG examination, and heart failure (HF) was classified as heart failure with reduced ejection fraction (HFrEF), mildly reduced ejection fraction (HFmEF), or preserved ejection fraction (HFpEF).

### 2.3 Statistical analysis

We performed data analysis using R software version 4.4.2 (R Foundation for Statistical Computing, Vienna, Austria). Continuous variables were presented as means with standard deviation (SDs) or with interquartile ranges (IQRs), depending on the normality test results. Normality was assessed via the Kolmogorov–Smirnov test, and homogeneity of variances was assessed via Levene's test. For continuous variables that followed a normal distribution and had homogeneity of variance, repeated measures two-way ANOVA was used for between-group comparisons. For non-normally distributed or heteroscedastic data, if normality and homogeneity of variance were achieved after logarithmic transformation, repeated measures two-way ANOVA was applied; otherwise, the Scheirer–Ray–Hare test was used. Categorical variables were described using frequencies and percentages and compared using the χ^2^ test or Fisher's exact test. First, we compared the baseline characteristics between the SGLT2i and non-SGLT2i groups, after which we conducted an analysis of the changes in coagulation parameters before and after treatment within each group. Furthermore, regression analysis was performed to evaluate the impact of SGLT2i treatment on coagulation function. For continuous dependent variables that followed a normal distribution, a linear mixed-effects model (LMM) was applied, whereas for non-normally distributed continuous dependent variables, a generalized linear mixed-effects model (GLMM) was used.

## 3 Results

### 3.1 Participant characteristics

**Figure 1:**
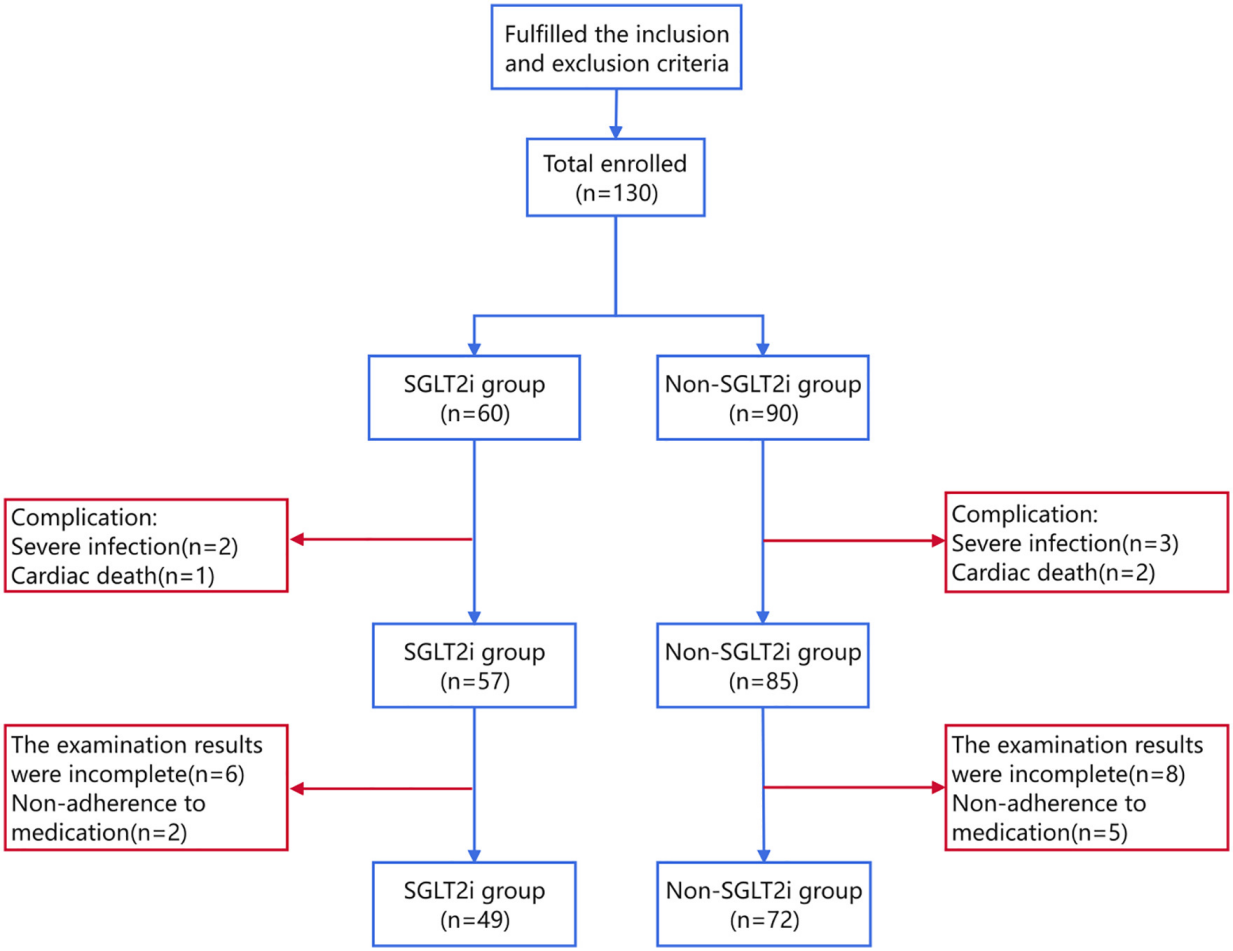
Participant selection flowchart.

A total of 121 patients were included in the current study, with 49 and 72 in the SGLT2i and non-SGLT2i groups, respectively. The participant selection flowchart is shown in Figure 1.

Comparisons of baseline clinical characteristics and medications between the two groups showed that only diuretic use had a statistically significant intergroup difference (Table 1). All other variables were comparable.

**Table 1:** Baseline clinical characteristic and medication comparisons between groups.

Characteristic	SGLT2i group (*n* = 49)	Non-SGLT2i group (*n* = 72)	*P*
Age, years, mean ± SD	60.06 ± 11.11	61.93 ± 11.42	0.373
Sex, *n* (%)			
Male, *n* (%)	33 (67.35%)	54 (75.00%)	0.476
Weight, kg, media (IQR)	71.69 ± 13.07	76.01 ± 16.71	0.121
Height, m, mean ± SD	167.73 ± 7.53	168.40 ± 7.86	0.642
BMI, kg/m^2^, mean ± SD	25.40 ± 3.83	26.68 ± 4.48	0.108
Blood pressure, mmHg, mean ± SD			
Systolic	134.08 ± 20.37	138.42 ± 22.30	0.279
Diastolic	79.06 ± 11.24	84.38 ± 13.60	0.026
Medications, *n* (%)			
Aspirin	49 (100.00%)	70 (97.22%)	0.653
Clopidogrel	22 (44.90%)	41 (56.94%)	0.264
Ticagrelor	26 (53.06%)	31 (43.06%)	0.370
Statins	48 (97.96%)	69 (95.83%)	0.901
Ezetimibe	13 (26.53%)	17 (26.61%)	0.880
ACEI or ARB	14 (28.57%)	28 (38.89%)	0.329
Beta-blockers	36 (73.47%)	51 (70.83%)	0.912
Diuretics	19 (39.78%)	14 (19.44%)	0.033
Metformin	18 (36.73%)	17 (23.61%)	0.174
Sulfonylureas	6 (12.24%)	14 (19.44%)	0.425
Insulin	11 (22.45%)	11 (15.28%)	0.445

Continuous data are presented as either mean with standard deviation (mean ± SD) or median with interquartile range (IQR), depending on the normality test results. Categorical variables are presented as frequency and percentage [*n* (%)].

SGLT2i, sodium/glucose cotransporter 2 inhibitor; ACEI, angiotensin-converting enzyme inhibitor; ARB, angiotensin II receptor blocker.

Baseline laboratory test and EKG result comparisons showed statistically significant intergroup differences in Glu and MCV levels (Table 2). Otherwise, all other measurements were similar between the groups.

**Table 2:** Baseline laboratory test and echocardiographic result comparisons between groups.

Test results	SGLT2i group (*n* = 49)	Non-SGLT2i group (*n* = 72)	*P*
Complete blood count			
RBC, 10^12^/L	4.56 ± 0.46	4.55 ± 0.54	0.923
HGB, g/L	139.80 ± 16.08	141.09 ± 15.85	0.663
Hct, %	41.14 ± 4.12	41.70 (38.30–44.63)	0.435
MCV, fl	90.33 ± 3.85	92.05 ± 4.92	0.042
PLT, 10^9^/L	222.00 (185.00–275.00)	226.00 (207.25–248.75)	0.659
PCT, %	0.23 (0.19–0.27)	0.22 (0.20–0.25)	0.477
MPV, fl	10.10 (9.10–11.10)	9.72 ± 1.05	0.083
Lipid profile			
LDL, mg/dl	0.17 ± 0.05	0.18 ± 0.06	0.329
HDL, mg/dl	0.05 (0.05–0.07)	0.06 (0.05–0.07)	0.592
ApoA1, mg/dl	116.00 (103.00–131.00)	122.50 (107.00–138.25)	0.143
ApoB, mg/dl	101.43 ± 31.45	96.50 (81.00–119.25)	0.704
TC, mg/dl	0.26 ± 0.07	0.25 (0.22–0.30)	0.584
TG, mg/dl	0.09 (0.07–0.13)	0.09 (0.06–0.12)	0.626
Lp(a), mg/dl	16.10 (10.00–25.60)	13.20 (6.08–26.25)	0.345
Blood chemistry			
ALT, U/L	25.00 (19.00–34.00)	30.00 (17.75–54.00)	0.128
AST, U/L	30.00 (20.00–91.00)	49.500 (19.00–125.00)	0.209
ChE, U/L	8,939.00 (7,533.00–10,362.00)	8,440.0 (7,314.50–9,515.00)	0.108
AKP, U/L	72.88 ± 18.39	70.00 (58.75–89.75)	0.720
Urea, mg/dl	0.33 (0.28–0.42)	0.31 (0.27–0.36)	0.154
UA, mg/dl	3.86 (3.17–4.93)	3.70 (3.07–4.57)	0.300
HbA1c, %	8.44 ± 1.47	7.85 (6.68–9.53)	0.229
Glu, mmol/L	8.00 (6.70–11.70)	6.70 (5.78–8.78)	0.022
eGFR, ml/min^−1^/1.73 m^−2^	99.02 ± 41.35	98.68 (80.24–123.95)	0.226
Cardiac function; *n* (%)			
HFrEF	6 (12.24%)	5 (6.94%)	0.501
HFmEF	12 (24.49%)	13 (18.06%)	0.529
HFpEF	17 (34.69%)	28 (38.89%)	0.782

Continuous data are presented as either mean with standard deviation or median with interquartile range, depending on the normality test results. Categorical variables are presented as frequency and percentage [*n* (%)].

SGLT2i, sodium/glucose cotransporter 2 inhibitor; RBC, red blood cell count; HGB, hemoglobin; Hct, hematocrit; MCV, mean corpuscular volume; PLT, total platelet count; PCT, plateletcrit; MPV, mean platelet volume; LDL, low-density lipoprotein; HDL, high-density lipoprotein; ApoA1, apolipoprotein A1; ApoB, apolipoprotein B; TC, total cholesterol; TG, triglycerides; Lp(a), lipoprotein a; ALT, alanine aminotransferase; AST, aspartate aminotransferase; ChE, cholinesterase; AKP, alkaline phosphatase; GGT, gamma-glutamyl transferase; UA, uric acid; HbA1c, hemoglobin A1c; Glu, glucose; eGFR, estimated glomerular filtration rate; HFrEF, heart failure with reduced ejection fraction; HFmEF, heart failure with mid-range ejection fraction; HFpEF, heart failure with preserved ejection fraction.

### 3.2 Differential analysis of coagulation profile changes

**Figure 2:**
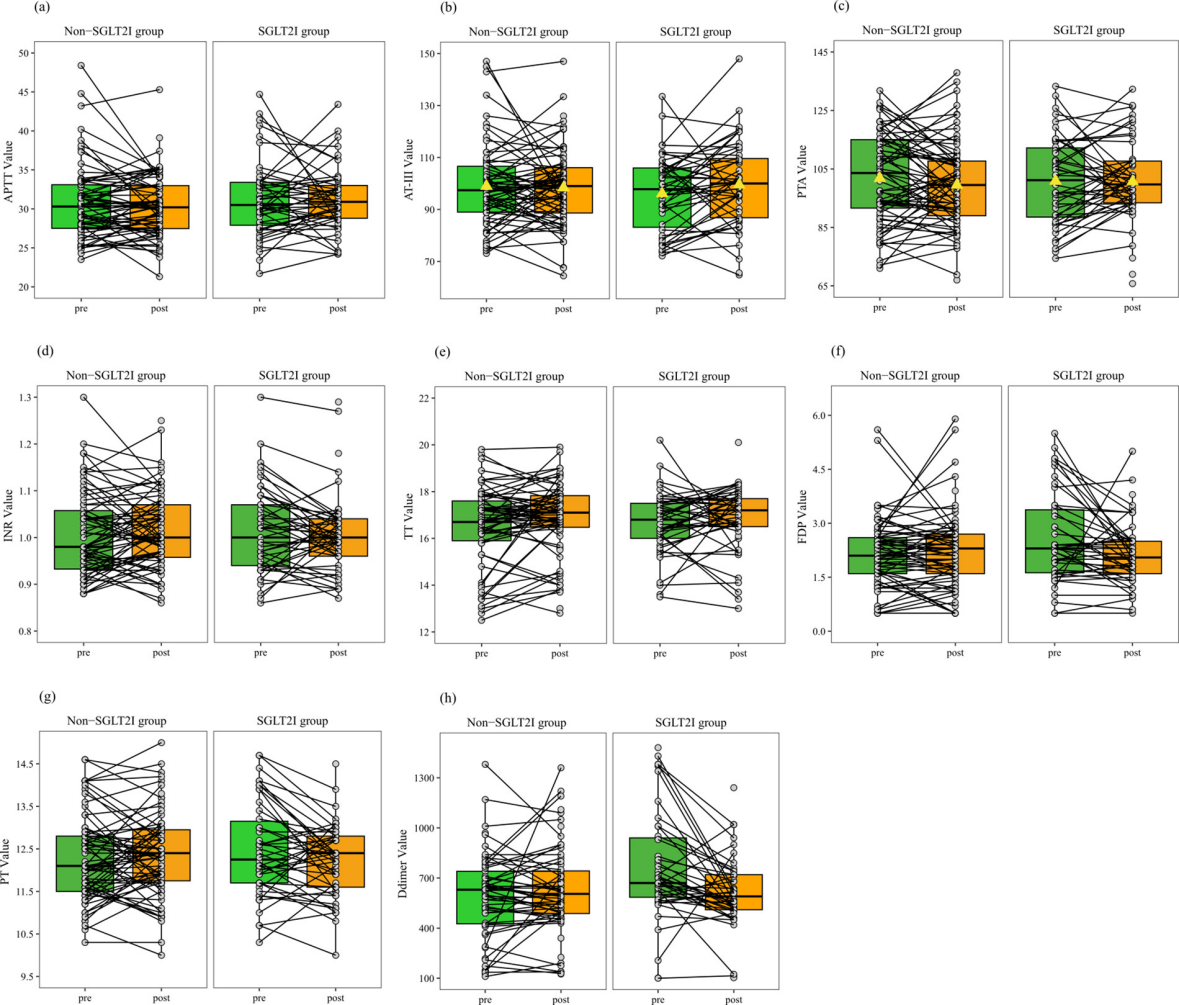
Paired line plots for stratified analysis comparing coagulation profiles before and after treatment between the SGLT2i and non-SGLT2i groups of patients stratified into the normal reference range subgroup. **(a)**, aPTT; **(b)**, AT-III; **(c)**, D-dimer; **(d)**, INR; **(e)**, PTA; **(f)**, TT; **(g)**, FDP; **(h)**, PT. **(b)** Unlike the non-SGLT2i group, the SGLT2i group exhibits an upward shift in the box plot, indicating a higher median after treatment. **(c)** Unlike the non-SGLT2i group, the SGLT2i group exhibits a downward shift in the box plot, suggesting a lower median after treatment. **(a, d–h)** Similar to the non-SGLT2i group, the SGLT2i group exhibits no significant change in the median or trend of the connected lines after treatment. **(b, c)** AT-III and PTA follow a normal distribution, with the mean represented by a triangle.

Since patients with T2DM and CAD often have abnormal coagulation profiles, we performed further analyses to examine coagulation profile changes following the SGLT2i treatment (Table 3, Figure 2).

**Table 3:** Post-treatment coagulation profile changes and differential analysis.

Coagulation profile	Group	Before	After	Time *P*	Group *P*	Time:Group *P*
aPTT, s	SGLT2i	30.70 (28.20–33.40)	30.90 (28.80–33.00)	0.123	0.387	0.786
	Non-SGLT2i	30.35 (27.65–33.45)	30.20 (27.48–32.98)			
AT-III, %	SGLT2i	94.10 ± 17.46	99.49 ± 16.48	0.268	0.634	0.026*
	Non-SGLT2i	99.04 ± 17.88	97.22 ± 17.53			
PTA, %	SGLT2i	98.32 ± 17.77	100.677 ± 14.13	0.412	0.954	0.292
	Non-SGLT2i	99.80 ± 17.84	99.51 ± 14.792			
INR	SGLT2i	1.02 (0.94–1.09)	1.00 (0.96–1.04)	0.286	0.907	0.276
	Non-SGLT2i	0.98 (0.94–1.07)	1.00 (0.96–1.07)			
TT, s	SGLT2i	17.00 (16.10–17.80)	17.20 (16.50–17.70)	0.649	0.852	0.584
	Non-SGLT2i	16.80 (16.13–17.83)	17.10 (16.48–17.83)			
FDP	SGLT2i	2.40 (1.70–3.50)	2.10 (1.60–2.50)	0.145	0.805	0.301
	Non-SGLT2i	2.35 (1.68–2.83)	2.30 (1.60–2.80)			
PT, s	SGLT2i	12.40 (11.70–13.60)	12.40 (11.60–12.90)	0.178	0.653	0.087
	Non-SGLT2i	12.10 (11.50–12.93)	12.40 (11.78–13.00)			
D-dimer, mg/L	SGLT2i	705.00 (600.00–1,010.00)	610.00 (510.00–740.00)	0.085	0.051*	0.075*
	Non-SGLT2i	625.00 (306.25–792.0)	595.00 (460.00–757.0)			

Continuous data are presented as either mean ± standard deviation or median (interquartile range), depending on the normality test results.

SGLT2i, sodium/glucose cotransporter 2 inhibitor; aPTT, activated partial thromboplastin time; AT-III, antithrombin III; PTA, prothrombin activity; INR, international normalized ratio; TT, thrombin time; FDPs, fibrin degradation products; PT, prothrombin time.

AT-III and PTA followed a normal distribution and had homogeneous variances; therefore, two-way repeated-measures ANOVA was used. TT, D-dimer, and FDP did not follow a normal distribution, and thus, the Scheirer–Ray–Hare Test was used. INR, aPTT, and PT were log-transformed to meet normality and homogeneity of variance, and the transformed data were analyzed using two-way repeated-measures ANOVA.

*P < 0.05.

After 30 days of treatment, the mean AT-III level in the SGLT2i group increased from a baseline mean of 94.10% to 99.49%, reflecting a 5.39% increase. In the non-SGLT2i group, the AT-III level decreased from 99.04% before treatment to 97.22% after treatment. Repeated measures two-way ANOVA indicated a significant interaction effect between time and group (*P* = 0.026), suggesting that AT-III changes differed between the groups. However, the overall effect of time alone was not significant (*P* = 0.268), indicating that SGLT2i treatment could have a potential upregulating effect on AT-III.

The median D-dimer level in the SGLT2i group decreased from a baseline value of 705.00 mg/L to 610.00 mg/L, reflecting a reduction of 95 mg/L. In the non-SGLT2i group, the median D-dimer level decreased from 625.00 mg/L before treatment to 595.00 mg/L after treatment. In terms of the magnitude of the reduction, the decrease in the non-SGLT2i group was smaller than that in the SGLT2i group. Repeated measures two-way ANOVA showed that although statistical significance was not reached, the interaction effect between time and group (*P* = 0.075) and the overall effect of time alone (*P* = 0.085) both showed trends toward significance, especially the group effect (*P* = 0.051). These findings suggest that SGLT2i treatment may reduce D-dimer levels, although the effect did not reach statistical significance and further analysis of potential impacts is needed.

The median PT in the SGLT2i group remained unchanged at 12.40 s. In the non-SGLT2i group, the median PT increased from 12.10 s before treatment to 12.40 s after treatment. Repeated measures two-way ANOVA showed no significant interaction effect between time and group (*P* = 0.087), nor significant differences in the overall time effect (*P* = 0.178) or group effect (*P* = 0.653). These findings suggested that while PT remained largely unchanged with SGLT2i treatment, the interaction effect between time and group (*P* = 0.087) was close to being significant, indicating the need for further analysis of the potential impact of group.

For the remaining coagulation parameters, including aPTT, PTA, INR, TT, and FDP, no statistically significant differences were observed between the SGLT2i and non-SGLT2i groups. Repeated measures two-way ANOVA showed no significant time effect, group effect, or time– group interaction for these indicators (all *P* > 0.05). These results suggested that SGLT2i treatment did not significantly alter these coagulation markers over the study period.

### 3.3 Regression analysis of coagulation profile

**Figure 3:**
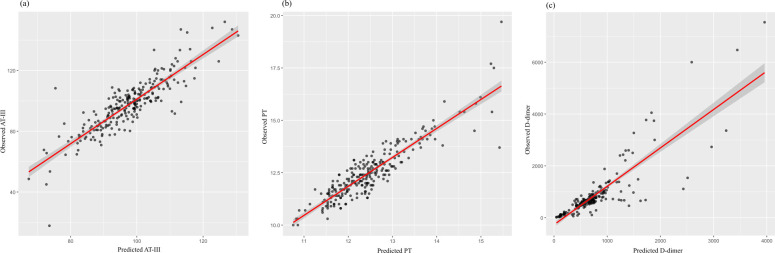
Scatter plots illustrating the correlation between the predicted and observed values of **(a)** antithrombin III (AT-III), **(b)** prothrombin time (PT), and **(c)** D-dimer. Each black dot represents an individual observation. The red line indicates the fitted regression line, with the shaded area representing the confidence interval. **(a, b)** The strong alignment along the diagonal suggests a good model fit for AT-III and PT predictions. **(c)** While most data points closely align with the regression line, a few extreme values suggest potential outliers or high variability in D-dimer predictions.

We conducted a regression analysis to evaluate the impact of SGLT2i treatment on AT-III, PT, and D-dimer. The results showed that the interaction between time and group significantly affected AT-III and D-dimer (*P* = 0.026 and *P* = 0.039, respectively). Specifically, the interaction coefficient for AT-III was positive (estimate = 7.207), indicating that the increase in AT-III levels over time was greater in the SGLT2i group than in the non-SGLT2i group. In contrast, the interaction coefficient for D-dimer was negative (estimate = −0.243), suggesting that D-dimer levels decreased more over time in the SGLT2i group than in the non-SGLT2i group. For PT, the estimated group effect of SGLT2i was 0.017, indicating that, on average, PT levels were slightly higher in the SGLT2i group than in the non-SGLT2i group, regardless of whether at baseline or one month after treatment. However, this difference was small and not statistically significant (*P* = 0.487). Additionally, the group effects of SGLT2i on AT-III and D-dimer were not statistically significant (Table 4, Figure 3).

**Table 4:** Regression analysis of coagulation profile.

Coagulation profile	Model	Fixed effects	Estimate	Std. Error	*t*	*P*
AT-III, %	Linear mixed model	Time:Group	7.207	3.202	2.251	0.026*
		Group	−4.940	3.225	−1.532	0.127
		Time	−1.824	2.037	−0.895	0.373
PT, s	Generalized linear mixed model	Time:Group	−0.022	0.013	−1.750	0.080
		Group	0.017	0.024	0.695	0.487
		Time	−0.002	0.008	−0.350	0.726
D-dimer, mg/L	Generalized linear mixed model	Time:Group	−0.243	0.118	−2.065	0.039*
		Group	0.331	0.210	1.572	0.116
		Time	−0.047	0.076	−0.615	0.539

Std. Error, standard error; AT-III, antithrombin III; PT, prothrombin time.

*P < 0.05.

**Figure 4:**
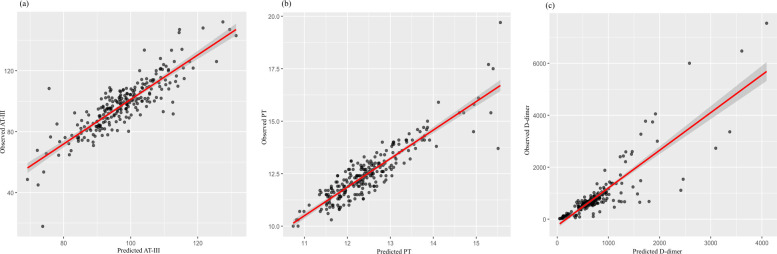
Scatter plots illustrating the correlation between predicted and observed values for **(a)** antithrombin III (AT-III), **(b)** prothrombin time (PT), and **(c)** D-dimer after adjusting for diuretic use (yes/no) in the regression models. Each black dot represents an individual observation. The red line denotes the fitted regression line, and the shaded area represents the 95% confidence interval. **(a, b)** The close alignment of data points along the diagonal line indicates a good model fit for AT-III and PT. **(c)** While most data points cluster around the regression line, a few extreme values suggest potential outliers or increased variability in D-dimer predictions.

Because the observed difference in diuretic use between the groups at baseline (Table 1) could potentially confound the results, we also performed regression analyses using linear or generalized linear models, with diuretic use (yes/no) included as a covariate. After adjusting for this potential confounder, the results remained consistent and robust (Table 5, Figure 4).

**Table 5:** Regression analysis of coagulation profiles with diuretic use included as a covariate.

Coagulation profile	Model	Fixed effects	Estimate	Std. Error	*t*	*P*
AT-III, %	Linear mixed model	Time:Group	7.526	3.226	2.333	0.021*
		Group	−6.067	3.234	−1.876	0.062
		Time	−1.905	2.051	−0.929	0.355
PT, s	Generalized linear mixed model	Time:Group	−0.020	0.013	−1.562	0.118
		Group	0.008	0.023	0.359	0.720
		Time	−0.003	0.008	−0.426	0.670
D-dimer, mg/L	Generalized linear mixed model	Time:Group	−0.211	0.105	−2.012	0.042*
		Group	0.265	0.212	1.248	0.212
		Time	−0.067	0.067	−1.004	0.316

Std. Error, standard error; AT-III, antithrombin III; PT, prothrombin time.

*P < 0.05.

## 4 Discussion

Patients with CAD and T2DM can be in hypercoagulable states and have a high risk for thrombotic formation, leading to serious complications. Herein, we showed for the first time that SGLT2is might exert anticoagulatory effects by increasing AT-III levels and prolonging PT in patients with CAD and T2DM. Our study results could provide a new evidence-based approach to alleviating hypercoagulable states and reducing thrombosis risk in these patients.

SGLT2is are a class of antihyperglycemic agents approved by the Food and Drug Administration (FDA) to treat patients with T2DM. Studies have shown that SGLT2 inhibitors could have CV protective functions, probably by reducing endothelial oxidative stress (OS) via inhibition of nicotinamide adenine dinucleotide phosphate (NADPH) oxidase activation (15, 16), decreasing cardiac preload and afterload via diuretic and natriuretic actions (6), alleviating sodium and calcium overload in cardiomyocytes by inhibiting sodium/hydrogen exchanger-1 (NHE-1) (17), reducing myocardial fibrosis (18), and modulating adipokines and inflammatory mediators (19–21). In the current study, we provided evidence to show that SGLT2is could mitigate the hypercoagulable state, which might contribute to their CV protective functions.

Baseline analysis before SGLT2i treatment revealed significant differences in Glu levels, diuretic use, and MCV between the SGLT2i and non-SGLT2i groups, indicating baseline disparities in metabolic status, medication use, and hematological parameters. In diabetic patients, blood Glu levels are closely related to liver and kidney function as well as blood cell morphology, while the use of diuretics can further influence blood and coagulation profiles. These baseline differences might imply the reliability of subsequent analyses.

To address potential bias arising from baseline differences, we accounted for these variables in subsequent analyses. Adjustments for confounding factors were implemented to mitigate the effects of baseline disparities and treatment-related influences, thereby enhancing the rigor and reliability of the results.

**Figure 5:**
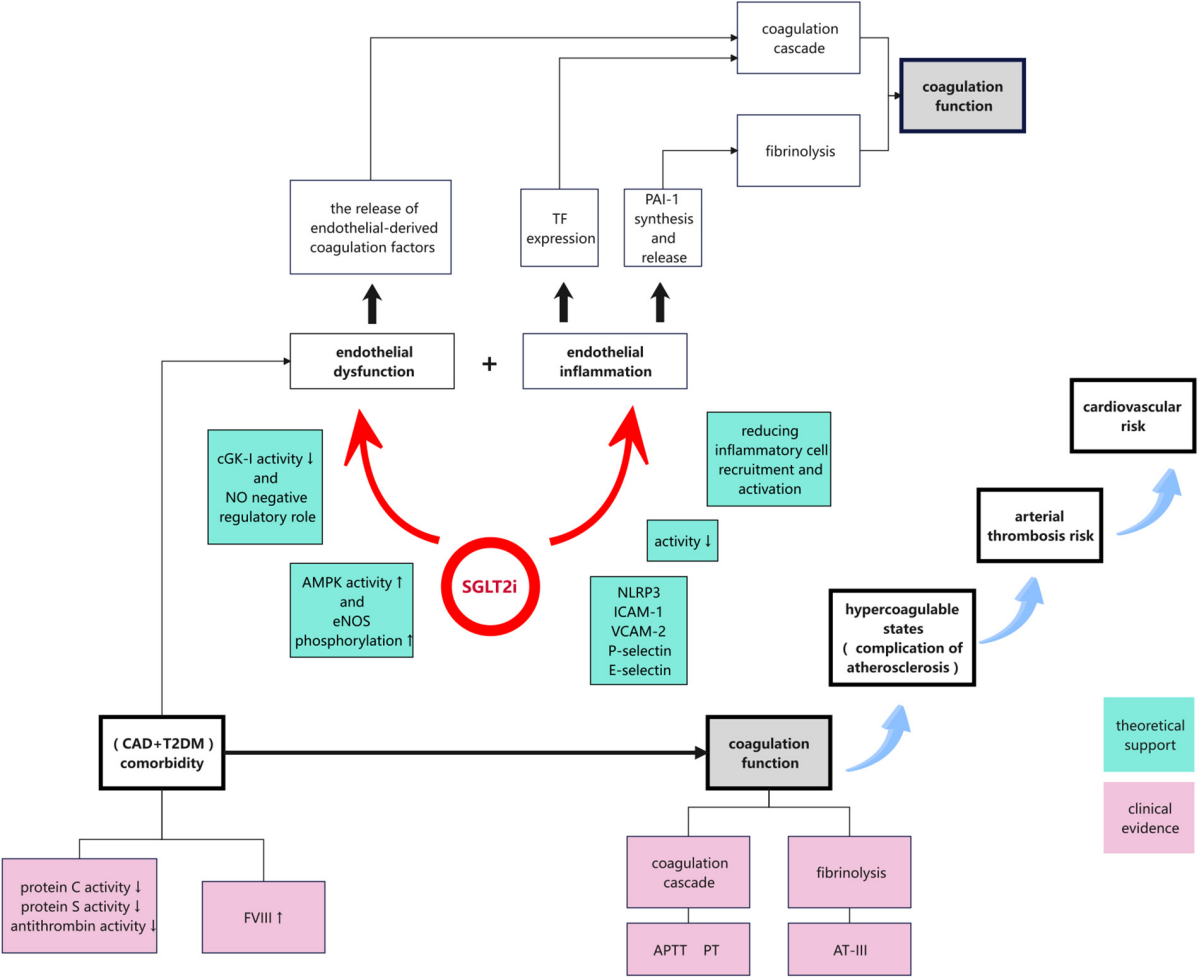
Proposed mechanisms by which SGLT2is modulated coagulation function in patients with CAD and T2DM via endothelial-function enhancement and anti-inflammatory effects. SGLT2is promoted nitric oxide production, suppressed the expression of inflammatory mediators, and reduced inflammatory-cell activation. These effects collectively mitigated the hypercoagulable state, decreased the risk of thrombosis, and could potentially reduce CV events in this high-risk patient population.

Patients receiving SGLT2i treatment exhibited a significant reduction in D-dimer levels, while the increase in AT-III levels approached statistical significance. Both D-dimer and AT-III are key markers of coagulation regulation. D-dimer is a degradation product of fibrin and serves as a crucial indicator of a hypercoagulable state, reflecting increased thrombus formation and fibrinolytic system activity. AT-III is a key anticoagulant protein that enhances the anticoagulation system by inhibiting activated coagulation factors. The increase in AT-III levels suggests enhanced anticoagulant activity. Additionally, we observed a potential increasing trend in PT, further indicating a hypocoagulable state. Taken together, these findings support the potential role of SGLT2is in modulating coagulation function (Figure 5). In contrast, no significant changes were detected in the non-SGLT2i group, highlighting the distinct impact of SGLT2is on coagulation function.

In our study, patients receiving SGLT2i treatment exhibited altered coagulation features that were characterized by a decreasing trend in D-dimer levels, but unchanged PT, suggesting that the extrinsic coagulation pathway was not significantly activated and that there were no thrombosis events. Notably, an increase in AT-III was also observed, indicating enhanced endogenous anticoagulant activity and a delayed intrinsic coagulation cascade. These findings suggest that SGLT2is might modulate the coagulation system to ultimately reduce thrombosis formation, possibly through the upregulation of endogenous anticoagulant mechanisms rather than by affecting the controlled activation of the extrinsic pathway.

In this study, although the observed coagulation profile changes caused by SGLT2is were relatively modest, they might still have indicated an effect of these drugs on coagulation function. While minor changes often have limited clinical effect in patients with a single disease, they can hold significant implications in those with multiple comorbidities. In patients with complicated disease backgrounds, subtle changes can reflect early pathological shifts and indicate underlying processes, and therefore they should not be overlooked. For example, Abdullah et al. reported that the hemostatic profiles of patients with CAD mirrored those of patients with venous thrombosis, with an elevated FVIII level contributing to a hypercoagulable state. In their study, linear-regression analysis showed a significant negative correlation between FVIII levels and aPTT in CAD patients (*R*^2^ = 10%, *P* < 0.0001), with each 1% increase in FVIII level associated with a 0.013-s reduction in aPTT [95% confidence interval (CI), −0.019 to −0.007] (2). An elevated FVIII level can accelerate activation of the intrinsic coagulation pathway and potentiate the extrinsic pathway, thereby increasing the risk of atherothrombosis. FVIII levels are markedly elevated in patients with comorbid CAD and T2DM, a rise that is inversely correlated with shortened aPTT. In the current study, after treatment with SGLT2is for 1 month, patients in the stratified normal-range subgroup had statistically significantly prolonged aPTT. This implied that SGLT2is exerted regulatory effects on coagulation function in patients with comorbidities, including comorbid CAD and T2DM.

Our findings suggested that SGLT2is might exert a unique anticoagulant regulatory effect in high-risk patients with both CAD and T2DM. Patients with multiple comorbidities often have chronic inflammation and hypercoagulability, requiring traditional anticoagulant therapy to carefully balance the risks of thrombosis and bleeding. By prolonging coagulation time and enhancing anticoagulant activity, SGLT2is might alleviate hypercoagulable states and reduce thrombosis risk without significantly increasing bleeding potential. This indicates that SGLT2is could serve as an adjunctive anticoagulant therapy in populations at high risk of MACEs and with multiple comorbidities, supporting more-personalized therapeutic strategies.

Furthermore, the impact of SGLT2is on coagulation function should not be considered an isolated finding, but instead as an integral component of their pleiotropic pharmacological profile. The mechanisms of these inhibitors include improved glycemic control, reduced vascular inflammation, altered coagulation pathways, and, more recently, anti-arrhythmic effects. Collectively, these effects suggest that SGLT2is may have broad therapeutic potential in patients with complex comorbidities. Recent studies have shown that SGLT2i use is associated with a reduced risk of atrial fibrillation and other arrhythmias, further supporting their potential role in rhythm stabilization (22, 23). Overall, these pleiotropic effects highlight the value of SGLT2is not only in metabolic and cardiovascular regulation, but also in the integrated management of thrombosis and arrhythmia. Future studies should explore the synergistic effects of SGLT2is and conventional anticoagulants in high-risk patient populations, with the goal of developing safer and more effective antithrombotic strategies.

Our study had several limitations, such as its retrospective study design and single-center nature. Additionally, the small sample size may limit the reliability of the analytical results. We studied coagulation profile changes after short-term (30-day) SGLT2i treatment. A long treatment duration might result in more-marked effects on coagulation profile. Many other factors, such as concomitant medication use and dietary patterns, could also affect coagulation function and coagulability state, but these factors could not be fully addressed in this retrospective study. Future multicenter studies with large sample sizes and long treatment durations are required to confirm our findings. Such studies will help assess whether the small changes in AT-III and D-dimer observed in the current study can be translated into significant therapeutic benefits, such as a reduction in thrombotic events or cardiovascular morbidity.

In conclusion, SGLT2is could prolong coagulation time and enhance anticoagulant activity, which affected coagulation function and contributed to CV protection in patients with CAD and T2DM. SGLT2is could be used to ameliorate hypercoagulable states and reduce thrombosis risk in such patients. Future research is warranted.

## Data Availability

The raw data supporting the conclusions of this article will be made available by the authors, without undue reservation.

## References

[1] Dal Canto E Ceriello A Rydén L Ferrini M Hansen TB Schnell O Diabetes as a cardiovascular risk factor: an overview of global trends of macro and micro vascular complications. Eur J Prev Cardiol. (2019) 26(2_suppl):25–32. 10.1177/2047487319878371 31722562

[2] Abdullah WZ Moufak SK Yusof Z Mohamad MS Kamarul IM. Shortened activated partial thromboplastin time, a hemostatic marker for hypercoagulable state during acute coronary event. Transl Res. (2010) 155(6):315–9. 10.1016/j.trsl.2010.02.001 20478546

[3] Ferrannini G Manca ML Magnoni M Andreotti F Andreini D Latini R Coronary artery disease and type 2 diabetes: a proteomic study. Diabetes Care. (2020) 43(4):843–51. 10.2337/dc19-1902 31988066

[4] May JE Moll S. How I treat unexplained arterial thrombosis. Blood. (2020) 136(13):1487–98. 10.1182/blood.2019000820 32584955

[5] Zinman B Wanner C Lachin JM Fitchett D Bluhmki E Hantel S Empagliflozin, cardiovascular outcomes, and mortality in type 2 diabetes. N Engl J Med. (2015) 373(22):2117–28. 10.1056/NEJMoa1504720 26378978

[6] McMurray JJV Solomon SD Inzucchi SE Køber L Kosiborod MN Martinez FA Dapagliflozin in patients with heart failure and reduced ejection fraction. N Engl J Med. (2019) 381(21):1995–2008. 10.1056/NEJMoa1911303 31535829

[7] Wiviott SD Raz I Bonaca MP Mosenzon O Kato ET Cahn A Dapagliflozin and cardiovascular outcomes in type 2 diabetes. N Engl J Med. (2019) 380(4):347–57. 10.1056/NEJMoa1812389 30415602

[8] Marx N Federici M Schütt K Müller-Wieland D Ajjan RA Antunes MJ 2023 ESC guidelines for the management of cardiovascular disease in patients with diabetes. Eur Heart J. (2023) 44(39):4043–140. 10.1093/eurheartj/ehad192 37622663

[9] Zhou L Cryan EV D'Andrea MR Belkowski S Conway BR Demarest KT. Human cardiomyocytes express high level of Na+/glucose cotransporter 1 (Sglt1). J Cell Biochem. (2003) 90(2):339–46. 10.1002/jcb.10631 14505350

[10] Aroor AR Das NA Carpenter AJ Habibi J Jia G Ramirez-Perez FI Glycemic control by the SGLT2 inhibitor empagliflozin decreases aortic stiffness, renal resistivity Index and kidney injury. Cardiovasc Diabetol. (2018) 17(1):108. 10.1186/s12933-018-0750-8 30060748 PMC6065158

[11] Sayour AA Korkmaz-Icöz S Loganathan S Ruppert M Sayour VN Oláh A Acute canagliflozin treatment protects against *in vivo* myocardial ischemia-reperfusion injury in non-diabetic male rats and enhances endothelium-dependent vasorelaxation. J Transl Med. (2019) 17(1):127. 10.1186/s12967-019-1881-8 30992077 PMC6469222

[12] Wan Z Wen W Ren K Zhou D Liu J Wu Y Involvement of NLRP3 inflammasome in the impacts of sodium and potassium on insulin resistance in normotensive Asians. Br J Nutr. (2018) 119(2):228–37. 10.1017/s0007114517002926 29359681

[13] Dmitrieva NI Burg MB. Elevated sodium and dehydration stimulate inflammatory signaling in endothelial cells and promote atherosclerosis. PLoS One. (2015) 10(6):e0128870. 10.1371/journal.pone.0128870 26042828 PMC4456159

[14] Shi L Wei X Luo J Tu L. SGLT2 inhibition, venous thrombolism, and death due to cardiac causes: a mediation Mendelian randomization study. Front Cardiovasc Med. (2024) 11:1339094. 10.3389/fcvm.2024.1339094 38803667 PMC11128626

[15] Khemais-Benkhiat S Belcastro E Idris-Khodja N Park SH Amoura L Abbas M Angiotensin II-induced redox-sensitive SGLT1 and 2 expression promotes high glucose-induced endothelial cell senescence. J Cell Mol Med. (2020) 24(3):2109–22. 10.1111/jcmm.14233 30929316 PMC7011151

[16] Miyata KN Lo CS Zhao S Liao MC Pang Y Chang SY Angiotensin ii up-regulates sodium-glucose co-transporter 2 expression and SGLT2 inhibitor attenuates Ang II-induced hypertensive renal injury in mice. Clin Sci (Lond). (2021) 135(7):943–61. 10.1042/cs20210094 33822013 PMC8131957

[17] Chen S Wang Q Bakker D Hu X Zhang L van der Made I Empagliflozin prevents heart failure through inhibition of the NHE1-NO pathway, independent of SGLT2. Basic Res Cardiol. (2024) 119:751–72. 10.1007/s00395-024-01067-9 39046464 PMC11461573

[18] Ma H Wu K Dong F Cai B Wu D Lu H. Effects of empagliflozin and dapagliflozin in alleviating cardiac fibrosis through SIRT6-mediated oxidative stress reduction. Sci Rep. (2024) 14:30764. 10.1038/s41598-024-30764-0 39730461 PMC11680569

[19] Morciano C Gugliandolo S Capece U Di Giuseppe G Mezza T Ciccarelli G SGLT2 inhibition and adipose tissue metabolism: current outlook and perspectives. Cardiovasc Diabetol. (2024) 23:39. 10.1186/s12933-024-02539-x 39702365 PMC11660748

[20] Xu Y Li L Ji Y Chen C Lu Y Zhang Y. Effects and mechanisms of SGLT2 inhibitors on the NLRP3 inflammasome, with a focus on atherosclerosis. Front Endocrinol (Lausanne). (2022) 13:992937. 10.3389/fendo.2022.992937 36589841 PMC9797675

[21] Mashayekhi M Safa BI Gonzalez MSC Kim SF Echouffo-Tcheugui JB. Systemic and organ-specific anti-inflammatory effects of sodium-glucose cotransporter-2 inhibitors. Trends Endocrinol Metab. (2024) 35(5):425–38. 10.1016/j.tem.2024.03.002 38423898 PMC11096060

[22] Duan HY Barajas-Martinez H Antzelevitch C Hu D. The potential anti-arrhythmic effect of SGLT2 inhibitors. Cardiovasc Diabetol. (2024) 23:252. 10.1186/s12933-024-02495-6 39010053 PMC11251349

[23] Mariani MV Lavalle C Palombi M Pierucci N Trivigno S D’Amato A SGLT2i reduce arrhythmic events in heart failure patients with cardiac implantable electronic devices. ESC Heart Fail [preprint]. (2025). 10.1002/ehf2.15223 39921334 PMC12055389

